# Longitudinal Effect of Hemoglobin Concentration With Incident Ischemic Heart Disease According to Hepatic Steatosis Status Among Koreans

**DOI:** 10.3389/fcvm.2021.677040

**Published:** 2021-05-28

**Authors:** Dong Hyuk Jung, Yong Jae Lee, Byoungjin Park

**Affiliations:** ^1^Department of Family Medicine, Yongin Severance Hospital, Yongin, South Korea; ^2^Department of Family Medicine, Yonsei University College of Medicine, Seoul, South Korea; ^3^Department of Family Medicine, Gangnam Severance Hospital, Seoul, South Korea

**Keywords:** hemoglobin, hepatic steatosis, cohort study, ischemic heart disease, risk factor, extrahepatic complications

## Abstract

**Background:** An increased hemoglobin (Hb) level may have detrimental effects on hepatic steatosis (HS) as well as cardiovascular disease (CVD). We investigated Hb's effect on incident ischemic heart disease (IHD) risk in the context of hepatic steatosis (HS).

**Methods:** We assessed 17,521 non-diabetic participants and retrospectively screened for IHD using the Korea National Health Insurance data. High Hb was defined as Hb levels ≥16.3 g/dL in men and 13.9 g/dL in women (>75th percentile). The participants were divided into five groups: reference (group 1), mild HS only (group 2), mild HS and high Hb (group 3), severe HS only (group 4), and severe HS and high Hb (group 5). We assessed hazard ratios (HRs) with 95% confidence intervals (CIs) for IHD using multivariate Cox proportional hazards regression models over 50 months from the baseline survey.

**Results:** During the follow-up period, 330 (1.9%) participants developed IHD (310 angina pectoris and 20 myocardial infarction). Compared with the reference group (group 1), the HRs for IHD were 1.04 (95% CI, 0.75–1.46) in group 2, 1.14 (95% CI, 0.70–1.85) in group 3, 1.58 (95% CI, 1.08–2.32) in group 4, and 1.79 (95% CI, 1.15–2.80) in group 5, after adjusting for IHD risk factors.

**Conclusions:** We found the combined effect of HS and Hb levels on the incidence of IHD.

## Introduction

Hepatic steatosis (HS) is an asymptomatic condition, but it is the most common liver disease in Western and developed Asian countries (1, 2). With an increase in high-calorie diet and obesity, triglyceride accumulates in the liver, resulting in inflammation and hepatic cellular damage, which progresses to steatohepatitis and fibrosis (3, 4).

Recently, extrahepatic complications have attracted growing interest as a risk factor for diabetes, atherosclerosis, and metabolic syndrome (5–7). In particular, HS's role in the pathogenesis of IHD has gained much interest. Epidemiological studies have reported a relationship between HSand the incidence of CVD events and mortality (8, 9). A previous study also showed that the severity of HS is dose-dependently related to atherosclerosis (10). However, these findings are more definite in patients with both hepatic manifestations and other risk factors such as advanced fibrosis and elevated hepatic enzymes (6). Thus, it is assumed that additional elements could modify the relationship between HS and IHD events. The serum hemoglobin (Hb) quantification can be performed using a simple blood test in a primary clinical setting and is essential when hematologic diseases, including anemia, are suspected (11). Previous studies have shown that Hb levels may be the modifying factors because an increased Hb level has detrimental effects on both IHD and non-alcoholic fatty liver disease (NAFLD). High Hb levels are closely related to body iron and ferritin stores (12). Elevated serum iron levels have been implicated in insulin resistance and cellular oxidative stress (13, 14). Thus, elevated Hb levels may be intricately associated with IHD and NAFLD. Despite the close interrelationship between Hb, HS, and IHD, only a few studies have investigated the interaction among them.

In this regard, we investigated the combined effect of HS and elevated Hb levels within the normal range on incident IHD risk in a large cohort of non-diabetic Korean adults using National Health Insurance data.

## Materials and Methods

### Study Design and Participants

**Figure 1:**
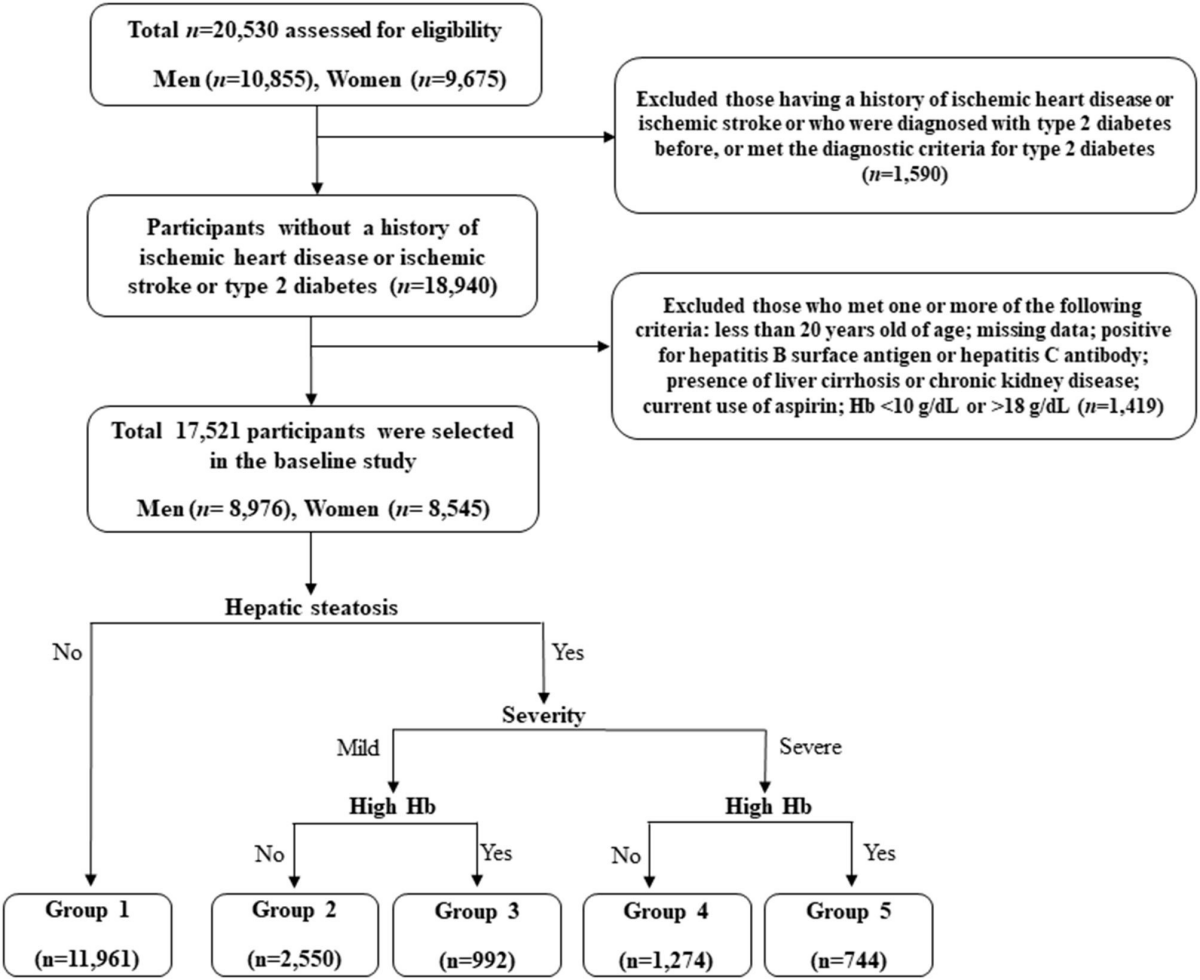
Flowchart for the selection of study participants.

This retrospective study is based on the Health Risk Assessment Study, aiming to explore surrogate markers for CVD among non-diabetic Korean adults. The study cohort consisted of 20,530 individuals who voluntarily visited the Health Promotion Centre of Gangnam Severance Hospital, Yonsei University College of Medicine, for regular health examinations between November 2006 and June 2010. Among the participants initially assessed, we excluded 1,590 (7.7%) subjects with a history of IHD or ischemic stroke, a previous diagnosis of type 2 diabetes, or a fasting plasma glucose level ≥126 mg/dL. Participants who met at least one of the following criteria were also excluded: <20 years of age; missing data; positive for hepatitis B surface antigen or hepatitis C antibody; presence of liver cirrhosis on abdominal ultrasonography; Hb levels below 10 g/dL or above 18 g/dL; presence of chronic kidney disease, defined based on either a reduced renal function or renal tissue damage with an eGFR <60 mL/min/1.73 m^2^ or proteinuria of ≥1+; current use of aspirin (*N* = 1,419). Following these exclusions, we included 17,521 participants (8,976 men and 8,545 women) in the final analysis (Figure 1).

### Data Collection

Each participant completed a questionnaire about their lifestyle and medical history. We obtained cigarette smoking status, alcohol consumption, and physical activity characteristics from the questionnaires. Smoking status was categorized as non-smoker and ex-smoker, or current smoker. Questions regarding alcohol intake included frequency of intake on a weekly basis. Regular alcohol drinking was defined as alcohol consumption ≥140 g per week. Participants were asked about the number of times per week they engaged in physical exercise, and regular exercise was defined as physical activity of moderate intensity exercising ≥ three times per week. Body weight and height were measured to the nearest 0.1 kg and 0.1 cm, respectively, in light indoor clothing without footwear. Body mass index (BMI) was calculated as a participant's weight divided by the height squared (kg/m^2^). Obesity was defined as BMI ≥ 25 kg/m^2^ according to Korean guideline. Systolic blood pressure and diastolic blood pressure were measured in the sitting position after 10 min using a standard mercury sphygmomanometer (Baumanometer, W.A. Baum Co Inc., Copiague, NY, USA) on the right arm. All blood samples were obtained from the antecubital vein after overnight fasting for 12 h. Hb levels were quantified using an automated blood cell counter (ADVIA 120, Bayer, NY, USA). Fasting plasma glucose, total cholesterol, triglyceride, HDL-cholesterol, aspartate aminotransferase (AST), and alanine aminotransferase (ALT) were measured by enzymatic methods using a Hitachi 7600 automated chemistry analyser (Hitachi Co., Tokyo, Japan). High-sensitivity C-reactive protein (hsCRP) concentrations were measured with a Roche/Hitachi 912 System (Roche Diagnostics, Indianapolis, IN, USA). Hypertension was defined as the current use of hypertension medication, a systolic blood pressure ≥ 140 mmHg, or diastolic blood pressure ≥ 90 mmHg. We calculated the HS index (HSI) score as follows: HSI score = 8 × ALT/AST ratio + BMI (kg/m^2^), (+ 2 for women).

### Abdominal Ultrasonography

Liver ultrasonography was performed using a 3.5-MHz transducer (HDI 5000, Philips, Bothell, USA) by experienced radiologists blinded to the laboratory and clinical data. Fatty liver was assessed semi-quantitatively and described as absent (grade 0), mild (grade 1), mild to moderate (grade 2), moderate (grade 3), moderate to severe (grade 4), or severe (grade 5), based on hepatorenal echo contrast, liver brightness, deep attenuation, and vascular blurring.

### Study Outcomes

To define outcomes, we linked a personal 13-digit identification number for each participant that was assigned using the Health Insurance Review and Assessment Data (HIRA), which is derived from the universal coverage system in Korea, from November 1, 2006 to December 31, 2010. The primary outcome was new-onset IHD, which comprised of angina pectoris (ICD-10 code I20) or acute myocardial infarction (ICD-10 code I21) occurring after study enrolment.

### Statistical Analysis

According to the fatty liver grade, the 17,521 participants were categorized into three groups: no HS and mild HS (grade 1–2) or severe HS (grade 3–5). High Hb was defined as Hb levels ≥ 16.3 g/dL in men and 13.9 g/dL in women (>75th percentile). To assess the combined effect of HS and Hb concentrations on incident IHD, we divided the study participants into five groups: no HS reference group, mild HS only group, mild HS and high Hb group, severe HS only group, and severe HS and high Hb group. The study population' baseline characteristics were compared among the groups using analysis of variance for continuous variables and chi-squared test for categorical variables. Kaplan–Meier curves were used to assess the cumulative incidence of IHD. The log-rank test was used to determine whether the distributions of the cumulative IHD incidence differed among groups. We used pairwise comparisons of receiver-operating characteristic (ROC) curves and concordance (C) statistic to assess the ability of a risk factor to predict IHD. After setting the first group as the reference group, the hazard ratios (HR) and 95% confidence intervals (CIs) for IHD were calculated using multivariate Cox proportional hazards regression models after adjusting for potential confounding variables. All analyses were performed using SAS version 9.4 (SAS Institute Inc., Cary, NC, USA). All statistical tests were two-sided, and statistical significance was set at *P* < 0.05.

## Results

**Figure 2:**
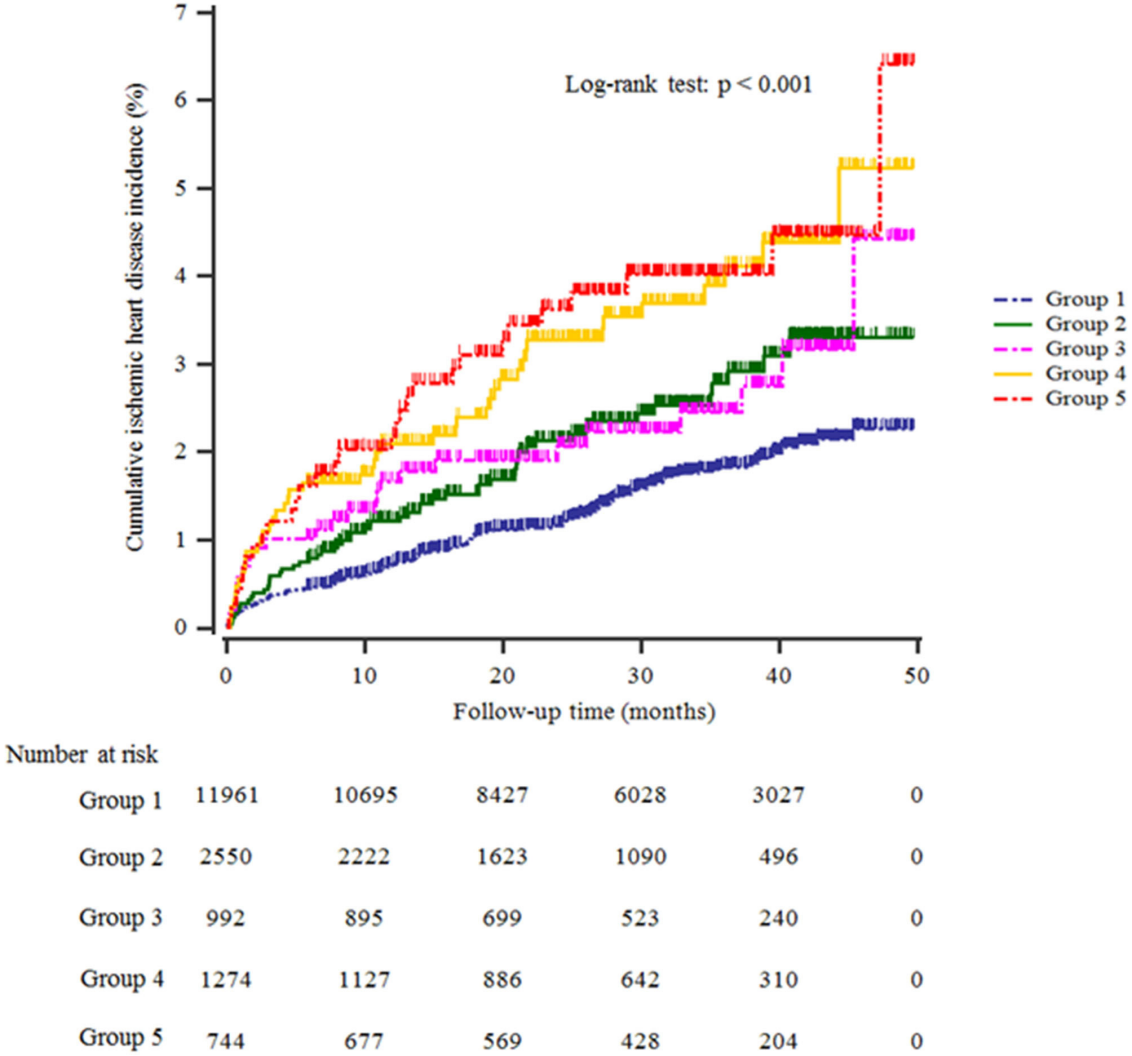
Kaplan–Meier plots indicating the cumulative probability of being diagnosed with ischemic heart disease after the baseline survey.

Table 1 shows the study population's baseline characteristics (*n* = 17,521; 8,976 men and 8,545 women) according to HS and Hb levels. The mean age and BMI were 44.7 ± 10.4 years and 23.3 ± 3.1 kg/m^2^. The mean Hb concentration was 14.4 ± 1.5 g/dL. The prevalence of severe HS was 11.5%. The mean values of mean arterial pressure, ALT, total cholesterol, and triglyceride levels were highest in the group with high HS and Hb levels (group 5). The most significant proportion of current smokers, alcohol drinkers, obesity, and hypertension was in group 5, while the proportion of individuals who participated in the regular exercise was lowest in group 4. Furthermore, group 5 showed the highest significant cumulative incidence of IHD over 50 months after the baseline survey (log-rank test, *P* < 0.001) (Figure 2).

**Table 1:** Baseline characteristics of the study population.

		**Group 1**	**Group 2**	**Group 3**	**Group 4**	**Group 5**		
**Characteristics**	**Overall (*n* = 17,521)**	**No hepatic steatosis (*n* = 11,961)**	**Mild hepatic steatosis only (*n* = 2,550)**	**Mild hepatic steatosis + high Hb (*n* = 992)**	**Severe hepatic steatosis only (*n* = 1,274)**	**Severe hepatic steatosis + high Hb (*n* = 744)**	***P*-value^a^**	***Post hoc*^b^**
Age (years)	44.7 ± 10.4	43.8 ± 10.6	47.2 ± 10.1	45.8 ± 9.5	46.2 ± 9.6	45.8 ± 10.5	<0.001	a,b,c,d,e,g
Male sex (%)	51.2	41.8	67.4	67.0	81.3	74.2	<0.001	–
BMI (kg/m^2^)	23.3 ± 3.1	22.2 ± 2.6	24.7 ± 2.5	25.2 ± 2.5	26.4 ± 2.9	26.6 ± 2.8	<0.001	a,b,c,d,e,f,g,h,i
Systolic BP (mmHg)	121.7 ± 15.4	118.8 ± 15.0	124.9 ± 14.5	128.8 ± 13.9	130.1 ± 14.1	132.1 ± 14.6	<0.001	a,b,c,d,e,f,g,i,j
Diastolic BP (mmHg)	75.9 ± 10.1	74.0 ± 9.7	78.0 ± 9.6	81.1 ± 9.2	81.5 ± 9.2	82.8 ± 9.4	<0.001	a,b,c,d,e,f,g,i,j
AST (IU/L)	21.5 ± 11.8	20.0 ± 10.0	21.9 ± 12.5	23.4 ± 8.4	28.3 ± 19.6	29.7 ± 14.9	<0.001	a,b,c,d,e,f,g,h,i
ALT (IU/L)	22.7 ± 21.8	18.5 ± 18.5	25.0 ± 20.1	29.1 ± 16.7	39.9 ± 33.0	43.8 ± 28.3	<0.001	a,b,c,d,e,f,g,h,i,j
GGT (IU/L)	31.2 ± 39.0	25.5 ± 32.2	37.0 ± 37.6	43.7 ± 40.0	50.5 ± 67.4	54.4 ± 49.1	<0.001	a,b,c,d,e,f,g,h,i
Hb (g/dL)	14.4 ± 1.5	14.1 ± 1.5	14.5 ± 1.3	16.0 ± 1.2	14.9 ± 1.1	16.3 ± 1.1	<0.001	a,b,c,d,e,f,g,h,i,j
FPG (mg/dl)	91.1 ± 9.8	89.2 ± 8.9	94.6 ± 9.7	93.9 ± 10.2	96.5 ± 10.6	96.8 ± 10.6	<0.001	a,b,c,d,f,g,h,i
Total cholesterol (mg/dl)	189.0 ± 33.5	183.9 ± 31.8	196.2 ± 34.5	203.0 ± 34.4	199.7 ± 32.7	209.2 ± 35.6	<0.001	a,b,c,d,e,f,g,i,j
Triglyceride (mg/dl)	122.7 ± 85.2	101.3 ± 57.2	151.4 ± 93.6	166.1 ± 90.9	186.7 ± 123.1	201.0 ± 160.0	<0.001	a,b,c,d,e,f,g,h,i,j
HDL-C (mg/dl)	53.6 ± 12.8	56.5 ± 12.8	48.7 ± 10.7	48.5 ± 10.4	44.8 ± 8.9	45.4 ± 8.7	<0.001	a,b,c,d,f,g,h,i
hsCRP (mg/L)	1.3 ± 3.6	1.1 ± 3.0	1.7 ± 5.6	1.7 ± 3.0	2.1 ± 3.7	2.0 ± 3.8	<0.001	a,b,c,d,f
Current smoker (%)	24.9	21.5	27.1	38.7	31.4	41.8	<0.001	–
Alcohol drinking (%)^c^	44.0	41.8	45.9	49.2	49.6	55.5	<0.001	–
Regular exercise (%)^d^	30.5	31.6	29.5	28.2	26.3	26.1	<0.001	–
Hypertension (%)	19.9	14.5	25.7	31.3	35.8	44.1	<0.001	
Obesity (%)	26.8	14.4	43.3	52.1	65.7	70.0	<0.001	
HSI score	32.2 ± 4.7	30.5 ± 3.7	34.2 ± 3.9	35.6 ± 4.0	37.7 ± 4.9	38.5 ± 4.6	<0.001	a,b,c,d,e,f,g,h,i,j

a *P-values were calculated using 1-way ANOVA or Pearson's chi-square test*.

b *Post hoc analysis using the Bonferroni method: a, Group 1 vs. Group 2; b, Group 1 vs. Group 3; c, Group 1 vs. Group 4; d, Group 1 vs. Group 5; e, Group 2 vs. Group 3; f, Group 2 vs. Group 4; g, Group 2 vs. Group 5; h, Group 3 vs. Group 4; i, Group 3 vs. Group 5, and j, Group 4 vs. Group 5*.

c *Alcohol intake ≥ 140 g/week*.

d *Moderate-intensity physical exercise ≥three times/week*.

*Hb, hemoglobin; BMI, body mass index; BP, blood pressure; ALT, alanine aminotransferase; AST, aspartate aminotransferase; GGT, γ-glutamyltransferase; FPG, fasting plasma glucose; HDL-C, high density-lipoprotein cholesterol; hsCRP, high-sensitivity C-reactive protein; HSI, hepatic steatosis index*.

**Figure 3:**
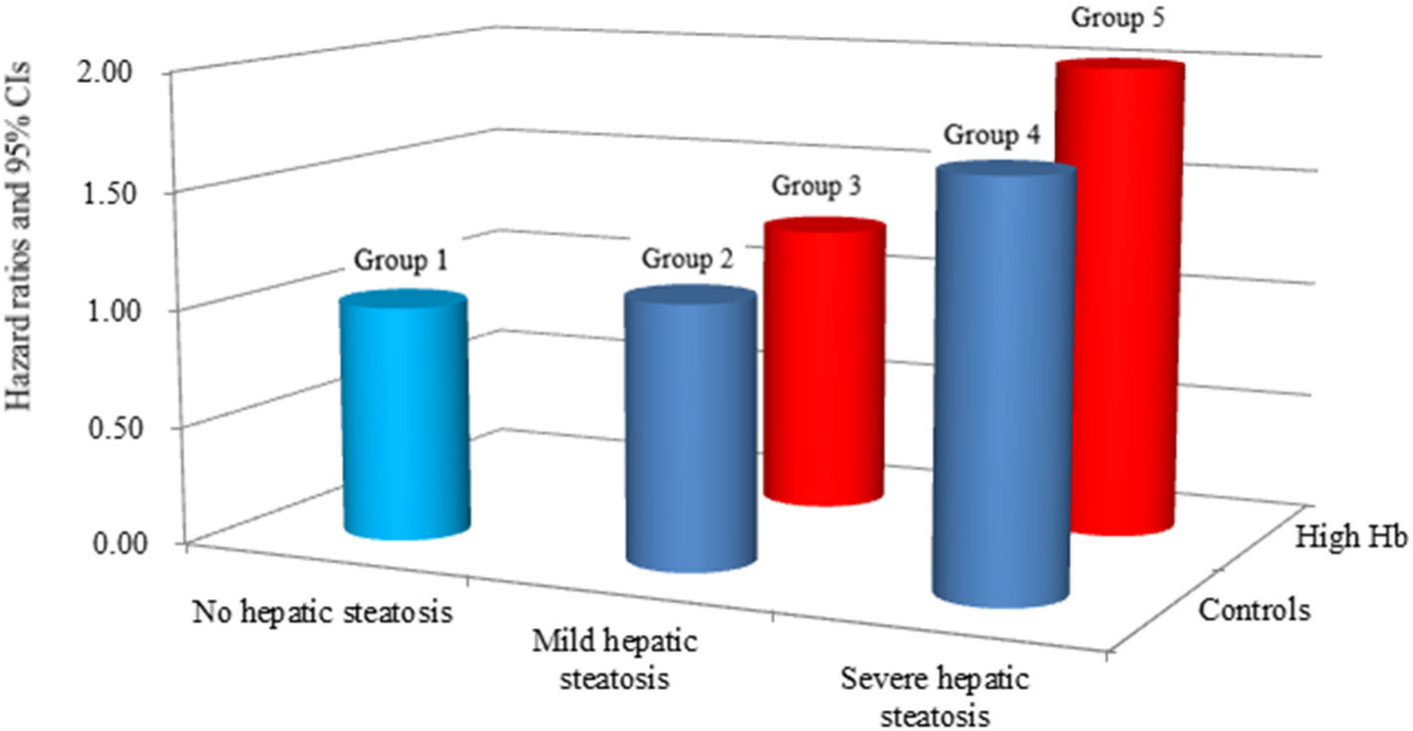
Hazard ratios for incident ischemic heart disease according to hepatic steatosis and hemoglobin after adjusting for age, sex, body mass index, smoking status, alcohol intake, and physical activity.

Table 2 shows the multivariate Cox proportional hazards regression results for the prediction of IHD according to HS and Hb levels. A total of 330 individuals (1.9%, 330/17,521) developed IHD during the follow-up period (310 angina pectoris and 20 myocardial infarction). Compared with the reference group (group 1), the HRs of IHD were 1.11 (95% CI, 0.80–1.98) in group 2, 1.23 (95% CI, 0.76–1.98) in group 3, 1.70 (95% CI, 1.16–2.48) in group 4, and 2.00 (95% CI, 1.29 to 3.10) in group 5 after adjusting for age, sex, BMI, smoking status, alcohol intake, and physical activity (Model 2, Figure 3). Similarly, these positive longitudinal associations were found after additionally adjusting for mean arterial pressure, fasting plasma glucose, total cholesterol, and hsCRP (Model 3). The corresponding adjusted HR for group 5 vs. group 1 was 1.79 (95% CI, 1.15–2.80).

**Table 2:** Hazard ratios and 95% confidence intervals for new-onset ischemic heart diseases according to hepatic steatosis and hemoglobin.

		**Group 1**	**Group 2**	**Group 3**	**Group 4**	**Group 5**	**P for trend**
		**No hepatic steatosis**	**Mild hepatic steatosis only**	**Mild hepatic steatosis + high Hb**	**Severe hepaticsteatosis only**	**Severe HS + high Hb**	
New cases of ischemic heart disease, n		177	56	24	44	29	
Mean follow-up, years		2.4 ± 1.1	2.2 ± 1.1	2.4 ± 1.1	2.4 ± 1.1	2.5 ± 1.1	
Pearson-years of follow-up		28,471	5,593	2,370	2,996	1,889	
Incidence rate/1,000 person -years		6.2	10.0	10.1	14.7	15.4	
Model 1	HR (95% CI)	1.00 (reference)	1.22 (0.90–1.65)	1.46 (0.95–2.23)	1.89 (1.35–2.65)	2.11 (1.42–3.13)	<0.001
	*P*-value		0.204	0.083	<0.001	<0.001	
Model 2	HR (95% CI)	1.00 (reference)	1.11 (0.80–1.98)	1.23 (0.76–1.98)	1.70 (1.16–2.48)	2.00 (1.29–3.10)	0.008
	*P*-value		0.534	0.404	0.006	0.001	
Model 3	HR (95% CI)	1.00 (reference)	1.11 (0.80–1.55)	1.23 (0.76–1.98)	1.75 (1.20–2.55)	2.00 (1.29–3.11)	0.005
	*P*-value		0.544	0.408	0.003	0.002	
Model 4	HR (95% CI)	1.00 (reference)	1.04 (0.75–1.46)	1.14 (0.70–1.85)	1.58 (1.08–2.32)	1.79 (1.15–2.80)	0.039
	*P*-value		0.806	0.591	0.019	0.010	

*Model 1: adjusted for age and sex*.

*Model 2: adjusted for age, sex, body mass index, smoking status, alcohol intake, and physical activity*.

*Model 3: adjusted for age, sex, obesity, smoking status, alcohol intake, physical activity, mean arterial blood pressure, fasting plasma glucose, total cholesterol, and high-sensitivity C-reactive protein level*.

*Model 4: adjusted for age, sex, body mass index, smoking status, alcohol intake, physical activity, mean arterial blood pressure, fasting plasma glucose, total cholesterol, and high-sensitivity C-reactive protein level*.

Using a pairwise comparison of ROC analyses of incident IHD, the C-index of the groups according to HS and Hb levels was similar to the C-index produced using the fatty liver grades and HSI (*P* = 0.123 and *P* = 0.829, respectively). The specificity of the groups according to HS and Hb levels for classifying IHD was higher than that of the HSI score, which was similar to that of the fatty liver grades (Table 3).

**Table 3:** Hepatic steatosis with hemoglobin vs. fatty liver grades and HIS score for predicting ischemic heart disease.

	**Pairwise comparison of C-index**			**Ability to classify IHD**			
	**Difference**	**95% CI**	***P*-value**	**Sensitivity (%)**	**Specificity (%)**	**C-index**	***P*-value**
Hepatic steatosis with Hb vs. fatty liver grade	0.003	−0.001 to 0.006	0.123				
Hepatic steatosis with Hb vs. HSI score	0.003	−0.025 to 0.031	0.829				
Fatty liver grade vs. HSI score	<0.001	−0.028 to 0.028	0.976				
Hepatic steatosis with Hb				46.4	68.5	0.584	<0.001
Fatty liver grade				46.4	68.5	0.581	<0.001
HSI score				69.1	46.1	0.581	<0.001

*Hb, hemoglobin; HSI, hepatic steatosis index; C-index, concordance index; IHD, ischemic heart disease*.

## Discussion

Among community-dwelling Korean adults without diabetes, we found that patients with HS were more likely to develop IHD than those without steatosis. Hepatic lipid accumulation was associated with dose-dependent IHD in this large-scale, cohort study that included a 50-month follow-up. This study also showed that HS with elevated Hb levels within the normal range is jointly related to the incidence of IHD in the general population. There could be a complex interaction between haematologic risk, metabolic abnormalities, and IHD.

A previous study reported that the presence of NAFLD at baseline, defined clinically using the calculated HSI, can more likely lead to the development of IHD (15). In this study, we calculated the probabilities of new-onset IHD for both HS, defined radiologically, and HSI, and we compared the C-index for each model. There was no statistically significant difference between the two predictive models, suggesting a little difference between imaging methods and the scoring system in grading HS based on the blood test. NAFLD comprises a spectrum of liver disease, ranging from simple steatosis to steatohepatitis and liver cirrhosis (16). Although liver biopsy is considered the gold standard for NAFLD assessment, it has limited applicability in clinical settings (17). Imaging methods, including ultrasonography and MRI, is widely used as diagnostic tools for HS in clinical practice (18). However, there is no correlation between the histologic and radiologic severity of HS. NAFLD can be characterized by hepatic tissue damage from the inflammation caused by HS (19).

In assessing patients' hepatic condition using radiologic methods, some serologic markers, representing hepatic damage, require particular attention. A previous study reported that ferritin, regarded as a measure of iron storage, correlates with Hb concentration (12). This means that high Hb levels could be another sign of high iron accumulation in human organs. Elevated ferritin levels are found in up to 30% of NAFLD patients, suggesting an association between elevated Hb levels and liver damage (20). The Korean Heart Study reported that high Hb levels increase the risk of IHD events (21). Iron accumulation, platelet count, and blood viscosity might explain why high Hb levels directly increase the development of IHD (21).

Although we could not determine the exact mechanism responsible for the complex interaction between Hb, HS, and IHD, several explanations for this interaction deserve consideration. Hb is an iron-containing protein, and iron accumulation can increase cardiovascular risk (22). Iron is a redox-active transitional metal and a potential catalyst in diverse cellular reactions resulting in reactive oxygen species (ROS) (23). ROS leads to endothelial tissue damage and metabolic disturbances, which are considered fundamental mechanisms for the development of IHD (24). In addition, ROS catalyzed by iron promotes low-density lipoprotein oxidation, and thus, they easily enter the arterial wall's inflammatory cells and lead to atherogenesis (25). Elevated Hb concentration contributes to increased blood viscosity, which increases peripheral resistance and decreases blood flow and perfusion (26). High Hb and hematocrit levels can induce platelet aggregation by releasing adenosine diphosphate (ADP) (27). A small population study showed that ADP-induced platelet aggregation was related to an increased incidence of coronary artery disease (28).

Previous data suggest that steatosis is related to cardiac arrhythmia and valvular heart disease, partly explaining the increased risk of IHD events in patients with steatosis (29, 30). Although HS with IHD is independent of elevated Hb levels, hepatotoxicity, induced by elevated Hb levels, may synergistically increase the risk of IHD development. A previous study has shown a bidirectional association between HS and elevated ferritin, represented by high Hb levels (31). HS is another clinical feature of insulin resistance. Insulin-induced downregulation of hepcidin increases iron absorption and accumulation in the liver and other organs (32). Conversely, iron accumulation may contribute to insulin resistance and hyperinsulinemia by interfering with hepatic insulin extraction (33). Furthermore, iron leads to highly toxic free radicals, such as the superoxide anion and hydroxide, through the Fenton reaction (23). Collectively, HS and elevated Hb levels are related to ROS and insulin resistance. They may share a common pathologic pathway, suggesting a possible synergistic action to the development of IHD.

Some strengths and limitations require careful consideration and may affect the interpretation of the present study results. A major strength of the work was that we conducted a cohort study using many Korean individuals linked to HIRA data, derived from the universal coverage system in Korea. As a result, there was a meager chance that the data was missing. Furthermore, this is the first study to investigate the combined effect of Hb levels and HS on the development of IHD, although these effects still require further confirmations. Another strength of our research is its large sample of 17,521 participants and its record of 330 IHD events over a median of 50 months follow-up.

This study had some limitations that should also be acknowledged. First, we could not assess the serum iron and ferritin levels from the HERAS-HIRA dataset. Further histochemical studies are needed because the relationship between Hb and tissue iron stores remains controversial. Second, although liver biopsy is the gold standard for diagnosing HS, we used imaging studies to define and grade HS. However, we also used a scoring system for the assessment of hepatic disease to decrease the bias in defining HS. Third, some individuals with diabetes may have been included in the participants because glycated hemoglobin A1c and 2-h oral glucose tolerance tests were not performed at the beginning of the study. Lastly, the HERAS-HIRA dataset assessed only newly developed IHD, not coronary angioplasty, myocardial resuscitation, or sudden death.

## Conclusions

This study showed that HS participants were more likely to develop IHD than those without steatosis, and hepatic lipid and triglyceride accumulation, represented by steatosis severity, are individually associated with dose-dependent IHD. Moreover, we confirmed the combined effects of HS and Hb levels on incident IHD.

## Data Availability Statement

The raw data supporting the conclusions of this article will be made available by the authors, without undue reservation.

## Ethics Statement

The studies involving human participants were reviewed and approved by The Institutional Review Board of Yonsei University College of Medicine. The patients/participants provided their written informed consent to participate in this study.

## Author Contributions

DJ, YL, and BP: study concept and design. YL and BP: acquisition, analysis, and interpretation of data and critical revision of the manuscript for important intellectual content. DJ: drafting of the manuscript. All authors contributed to the article and approved the submitted version.

## Conflict of Interest

The authors declare that the research was conducted in the absence of any commercial or financial relationships that could be construed as a potential conflict of interest.
